# Digestive Organs

**Published:** 1906-03-03

**Authors:** 


### DIGESTIVE ORGANS.

(*Continued from page* 355.)

**Bacteria in the Intestines.**—Among recent papers on normal intestinal bacteria may be mentioned that of Tarozzi,[8] who has investigated a group of organisms, occurring in man and other animals, which resemble in form the bacillus of tetanus. Though classed as anaërobes, these organisms can be cultivated in the presence of air by using specially prepared media, and thus further resemble the germs of tetanus and symptomatic anthrax. The spores live a considerable time if injected under the skin of animals, and the bacilli closely resemble those found in decomposing animal bodies. They are also found occasionally in the liver and spleen of old dogs. Tissier[9] finds that in new-born infants the bacteria vary in number in different parts of the gut, being few in the stomach, and increasing progressively to the rectum. In the duodenum, after a day or two, a sterilising action occurs, so that normally there are fewer bacteria here than in the stomach. Six varieties are described, of which the two commonest are the B. coli and the B. lactis aërogenes. As the gut is descended the bacteria become more anaërobic and more fermentative in character. In disease, Flexner and Holt,[10] aided by numerous other investigators, have shown that a large number of cases of infantile diarrhœa in America are due to the B. dysenteriæ, which organism has, however, been found occasionally in small numbers in the stools of healthy infants. It was not found, however, to be associated with the type of diarrhœa known as cholera infantum. The blood of infected infants agglutinated the bacillus in high dilutions. Serum treatment is disappointing in these cases. In adult cases of diarrhœa the serum-agglutination test should be used, as epidemics of dysentery are often preceded by simple diarrhœa. The results obtained by Jürgens and Dopter[11] show that, while it is difficult or impossible to isolate the bacillus from cases of simple diarrhœa occurring during or before an epidemic of dysentery, the agglutination test often gives valuable information. It usually appears after the diarrhœa has lasted about a fortnight, which is a considerably longer period than is necessary in typical dysentery. Negative results, however, are not of much value. By adopting this test the spread of an epidemic might very likely be checked in its early and often unrecognised stage. The investigation of the bacteria in the intestine naturally leads up to the question of intestinal antisepsis. Abbott[12] has recently revived the claims of the sulphocarbolates, but lays down certain rules for their administration. The bowels should first be emptied and peristalsis encouraged by moderate doses of calomel. These should be followed by a saline purge to increase the excretion of serum and thoroughly wash out the bowel. Then the sulphocarbolates can exert their action. The zinc salt is more astringent and antiseptic, the soda more antacid, and the lime salts more suitable to chronic and cachectic cases. Aldor[13] has investigated anew the possibility of high irrigation. He considers that arguments against the possibility of this process derived from experiments on the dead body are not valid. He has proved that a tube can be passed high up into the colon by skiagrams showing the tube *in situ*, and five years' experience have demonstrated to him the practical value of the measure.

**Appendicitis.**—A series of 5 cases diagnosed as appendicitis have recently been recorded by Maclachlan,[14] who draws from them some remarkable conclusions as to the ætiology of the condition. In one case the patient died a year later from phthisis, in another the patient's sister had died of phthisis, and in a third there had been a previous attack of pleurisy. In the remaining two cases no mention of a tuberculous taint is made. Maclachlan, however, regards the majority of cases of appendicitis as due to localised tuberculosis. The evidence does not appear to be particularly strong, as both conditions are sufficiently common to overlap in certain instances. Maclachlan's treatment consists in the early stages of drachm doses of magnesium sulphate every half-hour; later on he gives opium and aconite. He is much opposed to operation except when there is a localised abscess. Morris[15] has drawn attention to the fact that the onset of acute pneumonia may occasionally simulate appendicitis very closely, and his cases are well worth remembering now that early operation is often practised. His first case occurred in a young woman who for twenty-four hours had symptoms of an acute infection with abdominal rigidity, acute pain in the right iliac region, but no tenderness over the appendix, which could be felt to be adherent to the Fallopian tube. Operation proved the appendix normal, and on the next day the signs of pneumonia appeared for the first time in the chest. Even more misleading was the case of a boy of five who for four days had nausea, slight pyrexia, extreme abdominal rigidity, and pain in the right iliac region; operation proved the absence of peritonitis, and on the next day the physical signs appeared in the chest. Morris quotes further cases of his own and from the literature, and points out that the exclusion of appendicitis must be made by the temperature, which is usually too high, and by the absence of tenderness in the right iliac fossa. The pain no doubt is in these cases due to diaphragmatic pleurisy, and in children the frequent absence of the physical signs of pneumonia for some time should render the diagnosis of appendicitis less probable.

[8] Edin. Med. Chr. Jour., July, 1905, p. 87. [9] Ibid. [10] Pract., July, 1905, p. 87. [11] Lancet, Aug. 19, 1905, p. 545. [12] Med. Rec., Oct. 21, 1905, p. 683. [13] Ibid., Sept. 23, 1905, p. 504. [14] Caledonian Med. Jour., Oct., 1905, p. 213. [15] Med. Rec., July 1, 1905, p. 28.

